# Circulating osteocalcin is associated with time in range and other metrics assessed by continuous glucose monitoring in type 2 diabetes

**DOI:** 10.1186/s13098-022-00863-4

**Published:** 2022-08-04

**Authors:** Jun Liu, Yinghua Wei, Pu Zang, Wei Wang, Zhouqin Feng, Yanyu Yuan, Hui Zhou, Zhen Zhang, Haiyan Lei, Xinyi Yang, Jun Liu, Bin Lu, Jiaqing Shao

**Affiliations:** 1 Department of Endocrinology, Jinling Hospital, Southern Medical University, Nanjing, China; 2 Department of Endocrinology, School of Medicine, Jinling Hospital, Nanjing University, Nanjing, China; 3 Department of Endocrinology, Jinling Hospital, Nanjing Medical University, Nanjing, China

**Keywords:** Glycemic control, Time in range, Osteocalcin, Type 2 diabetes, HOMA-IS

## Abstract

**Background:**

Osteocalcin, a protein secreted mainly by mature osteoblasts, has been shown to be involved in glucose metabolism through various pathways. However, few studies has explored the association between osteocalcin and Time in range (TIR). Continuous glucose monitoring (CGM) -derived metrics, such as TIR and other indexes have been gradually and widely used in clinical practice to assess glucose fluctuations. The main purpose of this study was to investigate the correlation between osteocalcin and indexes from CGM in patients with type 2 diabetes mellitus (T2DM).

**Method:**

The total number of 376 patients with T2D were enrolled, all of them performed three consecutive days of monitoring. They were divided into four groups on account of the quartile of osteocalcin. Time in range, Time below range (TBR), Time above range(TAR) and measures of glycemic variability (GV) were assessed for analysing. After a 100 g standard steamed bread meal, blood glucose (Glu0h Glu0.5 h, Glu1h, Glu2h, GLu3h), C-peptide (Cp0h, Cp0.5 h, Cp1h, Cp2h, Cp3h), serum insulin (INS0h, INS0.5 h, INS1h, INS2h, INS3h) concentrations at different time points were obtained. HOMA-IS, HOMA-βwas calculated to evaluate insulin sensitivity and insulin secreting of the participants.

**Results:**

Patients with higher osteocalcin level had higher TIR (P < 0.05). Spearman correlation analysis showed that osteocalcin was positively correlated with TBR (although the P value for TBR was greater than 0.05) (r = 0.227, P < 0.001 r = 0.068, P = 0.189) and negatively correlated with TAR (− 0.229, P < 0.001). Similarly, there was a negative correlation between osteocalcin and glycemic variability (GV) indicators, including SD, MBG, MODD, ADDR, and MAGE (P value of MAGE > 0.05). Multiple stepwise regression showed that osteocalcin was an independent contributor to TIR, TAR and HOMA-IS.

**Conclusion:**

Circulating osteocalcin is positively correlated with TIR and negatively correlated with MODD, ADDR, and MAGE. Osteocalcin may have a beneficial impact on glucose homeostasis in T2DM patients.

## Background

In the last few decades, the number of people with diabetes has increased from 151 to 463 million by 2020 [1], and diabetes has become the ninth leading cause of death worldwide [2]. Recent diabetes treatment guidelines emphasize individualized treatment in the management of diabetes patients. As a plausible and common biomarker, HBA1c is a gold standard for assessing long-term glycemic control over the past 2 to 3 months [3, 4]. A series of studies have demonstrated that HBA1c plays a prominent role in the management of diabetes [5, 6]. However, the accuracy of HbA1C measurements can be affected by various clinical conditions [7, 8]. Moreover, HbA1C does not reflect blood glucose fluctuations, and thus fail to prevent potential harmful hyperglycemic or hypoglycemic events [9, 10]. In contrast, continuous glucose monitoring (CGM) provides a more accurate picture of glycemic variability by measuring glucose concentrations in the interstitium continuously for several days. A standardized CGM report was recommended internationally in 2017 [7]. The parameters derived from CGM, including TIR, TBR, TAR, SD, MBG, MODD, and ADDR [11], are widely used to assess glycemic variability in clinical. Furthermore, TIR was recommended as one of the target indicators for glycemic control assessment by the ADA recently [12].

Osteocalcin, a bone γ-carboxyglutamate (GLA) protein encoded by the human osteocalcin gene BGLAP, is mainly produced by osteoblasts [13, 14]. Osteocalcin was originally considered to act only in skeletal cells when it was first discovered in the 1976s [15]. Lee identified a novel function of osteocalcin in Ocn-/- mice in 2007, indicating that osteocalcin may be involved in regulating energy metabolism [16]. Although numerous studies have explored the relationship between osteocalcin and glucose metabolism since then, the results remains are still controversial. A cohort study found that osteocalcin levels in the HbA1c ≥ 9% group were significantly lower than those in the HbA1c < 7% group [17]. In contrast, another study showed that osteocalcin levels decreased after daily calcium supplements for one year in older community-dwelling women, whereas HbA1c did not change [18]. On the other hand, few studies have explored the correlation between osteocalcin and glucose variability, the effects of osteocalcin on glucose fluctuations remain unclear. The purpose of this study is to investigate the correlation between steocalcin and TIR and other parameters of glycemic variability from CGM in T2DM patients.

## Study design and methodology

### Research Subjects

A total of 376 participants were recruited from the Department of Endocrinology in Jinling Hospital of Southern Medical University according to the diagnostic criteria published by the WHO in 1999 [19]. Inclusion criteria were age ≥ 18 years and having a stable glucose- lowering treatment within the last three months. Individuals who met one of the following criteria were excluded: hyperglycemic hyperosmolar state or severe hypoglycemic events within the past 3 months; diabetic ketoacidosis; history of cancer and psychiatric disease; severe hepatic or renal dysfunction; comorbid thyroid or parathyroid disease; diagnosis of osteoporosis; and treatment with medications that can affect bone and calcium metabolism, such as vitamin D, calcitonin, bisphosphonates, or estrogens. The local ethics committee approved the study in accordance with the Declaration of Helsinki.

### Anthropometric and biochemical assessment

Basic characteristics including age composition, gender, duration of diabetes mellitus, height and weight were collected. Systolic blood pressure (SBP) and diastolic blood pressure (DBP) were measured with an accurate sphygmomanometer, and BMI was obtained according to an international formula. After an all-night fast, venous blood samples were drawn from participants by a professional the day before the CGM was performed. HbA1c was measured by high performance liquid chromatography (HLC-723G8 automated glycated hemoglobin analyzer, TOSOH, Japan). Biochemical indicators were measured by an automated biochemical analyzer (7600 series automated analyzer, Hitachi, Japan). Circulating osteocalcin levels, insulin and C-peptide concentrations were obtained by chemiluminescent immunoassay (IMMULITE 2000 XPi, Siemens, Germany). HOMA-IS and HOMA-β were calculated according to the standard formula {HOMA-β = 20 × fasting insulin (FINS mIU/L)/[fasting serum glucose (FPG mmol/L)- 3.5], HOMA-IS = 22.5/[FPG (mmol/L) × FINS (mIU/L)]}.

### CGM parameters

Patients were tested by CGM (Meiqi) for three consecutive days (all participants were informed about the system and its safety by a regular professional before participating in the experiment). Raw blood glucose data were detected every three minutes and were not visible to the patients. Oxford's EasyGV version 9.0R2 was used to assess glycemic control metrics. Each individual was taught to avoid any strenuous activity that could affect blood glucose. TIR was defined as the percentage of time that blood glucose level remained between 3.9 and 10 mmol/L throughout the day, TBR represented the percentage of time that blood glucose was < 3.9 mmol/L in a 24-h period, and TAR was the proportion of time that exceeded 10.0 mmol/L during the day [7]. Glycemic control indicators such as standard deviation (SD), coefficient of variation of glucose (CV), low glycemic index (LBGI), high glycemic index (HBGI), mean amplitude of glucose excursion (MAGE), mean daily risk range (ADDR), and mean daily difference (MODD) were calculated and analyzed.

### Statistical analysis

Patients were classified as G1 (osteocalcin < 10.68 ng/ml), G2 (10.68 ng/ml≦osteocalcin < 13.72 ng/ml), G3 (13.72 ng/ml≦osteocalcin < 17.30 ng/ml) and G4 (osteocalcin ≧17.30 ng/ml) according to the quartiles of osteocalcin levels. Data are expressed as mean ± SD, median [25% 75%]. Categorical variables were tested by chi-square test. Regarding continuous and normally distributed variables, one-way ANOVA was used to assess trends in each group, while Kruskal–Wallis H-test was used for abnormalities. We performed Spearman rank correlation to assess the association between osteocalcin and other variables. After adjusting for age, duration of diabetes, gender, BMI, SBP, DBP, TG, TC and other indicators, multivariate linear regression analysis was used to examine the independent association of osteocalcin with CGM parameters. We considered P < 0.05 as statistically significant. SPSS 25.0 software was used for analysis.

## Results

### Comparison of clinical characteristics between groups

**Fig. 1:**
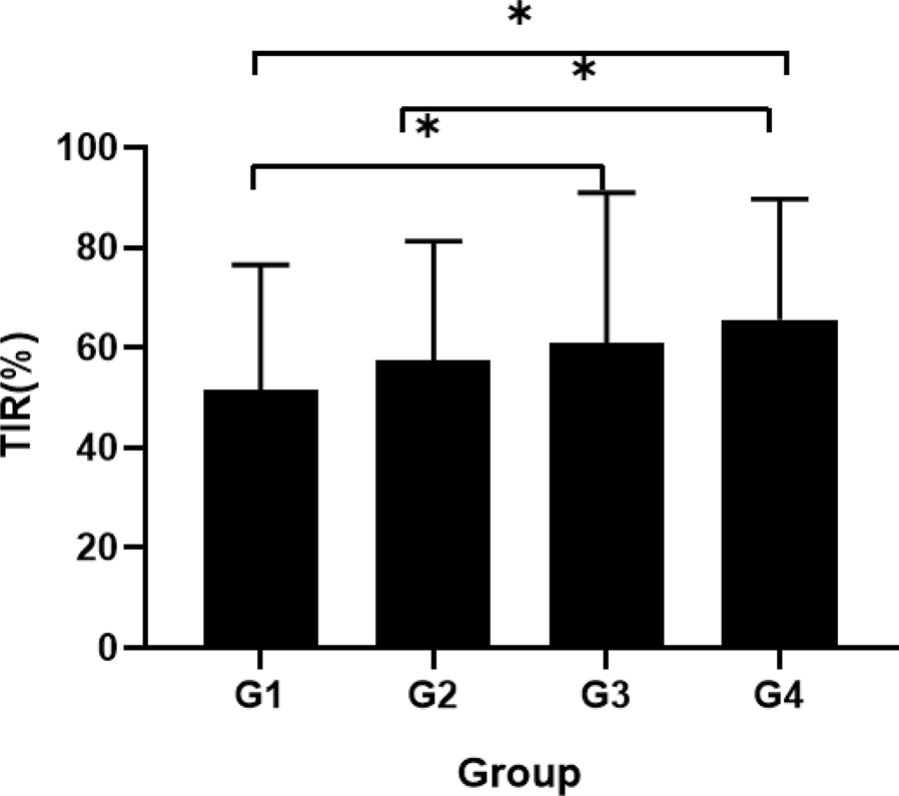
TIR of each group by quartile of osteocalcin levels (G1:osteocalcin < 10.68 ng/ml, G2:10.68 ng/ml≦osteocalcin < 13.72 ng/ml, G3:13.72 ng/ml≦osteocalcin < 17.30 ng/ml, G4:osteocalcin≧17.30 ng/ml). Kruskal–Wallis H test was applied to show statistical significance of comparison between groups (n = 94 per group) *p = 0.001

In our study, 376 patients with T2DM (235 males and 141 females) were divided into four groups according to the quartiles of osteocalcin (Table 1). The osteocalcin concentrations in groups 1 to 4 were 8.73 (7.50 9.82) ng/ml, 12.38 (11.43 13.19) ng/ml, 15.10 (14.51 16.00) ng/ml, and 21.20 (18.70 26.27) ng/ml, respectively. The TIR of each group is shown in Fig. 1. There were no statistically significant differences between the groups in terms of gender composition, age, weight, BMI, duration of diabetes or blood pressure (P > 0.05, respectively). Similarly, there were no differences between groups in biochemical measurements, such as TC, TG, serum K + , serum Ca + , creatinine and total vitamin D (Table 1).

**Table 1:** Characteristics of study participants by quartiles of Osteocalcin

Variables	G1	G2	G3	G4	P
Number	94	94	94	94	
Male/Female	M 60 F 34	M 62 F 32	M 59 F 35	M 54 F 40	
Osteocalcin (ng/ml)	8.73 (7.50 9.82)	12.38 (11.43 13.19)*	15.10 (14.51 16.00)*#	21.20 (18.70 26.27)*#✱	< 0.001
TBR (%)	0.00 (0.00 0.00)	0.00 (0.00 0.00)	0.00 (0.00 0.04)	0.00 (0.00 0.47)	0.71
TIR (%)	53.94 (34.01 70.93)	58.43 (40.47 76.11)	68.64 (34.76 86.93)*	72.20 (45.55 85.93)*#	0.001
TAR (%)	45.24 (29.07 64.29)	41.57 (23.15 59.44)	30.86 (13.03 62.84)*	26.74 (13.32 53.07)*#	0.001
Age (years)	55.68 ± 11.43	56.49 ± 11.13	53.27 ± 3.80	55.30 ± 12.84	0.403
Weight (Kg)	70.41 ± 12.30	70.25 ± 12.20	71.7 ± 12.63	68.15 ± 12.69	0.288
High (m)	1.66 ± 0.08	1.66 ± 0.08	1.68 ± 0.09	1.67 ± 0.09	0.467
BMI (kg/m2)	25.37 ± 3.62	25.03 ± 3.21	25.08 ± 3.27	24.74 ± 3.70	0.669
Creatinine (umol/L)	57.16 ± 16.34	58.02 ± 13.35	56.27 ± 14.87	59.24 ± 17.45	0.615
Duration (years)	7.00 (2.00 15.00)	8.00 (2.75 15.00)	6.00 (2.00 12.00)	6.00 (1.00 11.00)	0.419
SBP (mmHg)	130.00 (127.75 145.00)	130 (124.75 140.00)	130 (129.50 140.00)	130.00 (120.00 138.50)	0.499
DBP (mmHg)	78.00 (75.00 86.25)	79.00 (75.00 85.25)	79.00 (75.00 87.50)	78.00 (74.00 82.25)	0.705
K (mmol/L)	3.82 ± 0.37	3.8 ± 0.33	3.86 ± 0.32	3.84 ± 0.29	0.581
Ca (mmol/L)	2.21 ± 0.12	2.24 ± 0.12	2.21 ± 0.10	2.19 ± 0.11	0.053
TC (mmol/L)	4.42 ± 1.14	4.63 ± 1.07	4.56 ± 0.95	4.71 ± 1.04	0.297
TG (mmol/L)	1.53 (1.10 2.32)	1.61 (1.12 2.28)	1.50 (1.10 2.35)	1.44 (1.01 1.98)	0.602
HbA1C (%)	9.25 ± 1.73	9.00 ± 1.98	8.62 ± 2.21*	8.49 ± 1.94*	0.045
Vit D (ng/ml)	24.11 ± 6.84	25.56 ± 6.64	25.15 ± 6.86	23.73 ± 6.27	0.218
MBG (mmol⁄ L)	10.35 ± 2.02	9.89 ± 2.12	9.53 ± 2.49*	9.21 ± 1.79*#	0.002
SD	2.86 ± 1.01	2.59 ± 0.99	2.35 ± 0.88*	2.44 ± 0.97*	0.008
CV	0.28 ± 0.08	0.28 ± 0.10	0.24 ± 0.06*#	0.26 ± 0.08✱	< 0.001
LBGI	0.37 (0.00 1.74)	0.50 (0.05 1.50)	0.37 (0.01 1.03)	0.74 (0.21 1.81)*✱	0.043
HBGI	10.26 (7.08 14.40)	9.61 (6.07 13.82)	7.13 (3.61 13.82)*	6.74 (3.75 12.87)*#	0.002
MAGE (mmol⁄ L)	5.30 ± 2.03	4.78 ± 1.76	4.75 ± 1.72	4.89 ± 1.69	0.171
ADDR (mmol⁄ L)	27.27 (20.61 37.24)	25.31 (18.53 32.40)	20.84 (13.91 32.64)*	21.29 (13.15 31.86)*	0.001
MODD (mmol⁄ L)	2.44 (1.68 3.37)	2.38 (1.76 3.52)	2.07 (1.35 2.56)*#	2.05 (1.43 3.16)	0.003

Data are presented as means ± SD, median (25% and 75%interquartiles), and count (percentages) according to characteristics of the distribution. Between-group, comparisons were conducted by One-way ANOVA, Kruskal- Wallis H test, and the chi-squared test

BMI: body mass index, TC: total cholesterol, TG: triglycerides, SBP: systolic blood pressure, DBP: diastolic blood pressure, HbA1c: hemoglobin A1C, TIR: time in range, TBR: time below range, TAR: time above range, CV: coefficient variation, SD: standard deviation, LBGI: low blood glucose index, HBGI: high blood glucose index, MAGE: mean amplitude of glucose excursions, ADDR: average daily danger range, MODD: mean of daily differences

^*^Significant difference with group 1(P < 0.05)

^#^Significant difference with group 2(P < 0.05)

^✱^Significant difference with group 3(P < 0.05)

### Correlation of serum osteocalcin and glucose indexes

Patients with G3 and G4 tended to have lower TAR and HbA1C levels, as well as MBG, SD, CV, HBGI, ADDR and MODD compared to G1 (P < 0.05). In contrast, the TIR and LBGI were lower in G1. Positive correlations were found between osteocalcin and TIR and HOMA-IS (r = 0.227, 0.192 P < 0.001), negative correlations were found between osteocalcin and TAR and HbA1C, while the relationship between osteocalcin and HOMA-βwas not significant (P = 0.801). Similarly, blood glucose at INS0h, INS0.5 h and all time points were inversely correlated with circulating osteocalcin (Table 2). As for the glycemic variability indices, HBGI, SD, CV, MBG, MODD, MAGE and ADDR all decreased with increasing serum osteocalcin levels.

**Table 2:** Spearman Partial Correlation Among Osteocalcin and Selected CGM Metrics

Variables	Osteocalcin
TIR	r = 0.227
	P < 0.001
TBR	r = 0.068
	P = 0.189
TAR	r = − 0.229
	P < 0.001
HbA1C	r = − 0.143
	P = 0.006
INS0h	r = − 0.169
	P = 0.001
INS0.5 h	r = − 0.115
	P = 0.03
INS1h	r = − 0.081
	P = 0.128
INS2h	r = − 0.016
	P = 0.762
INS3h	r = − 0.027
	P = 0.603
Cp0h	r = − 0.064
	P = 0.219
Cp0.5 h	r = -0.039
	P = 0.448
Cp1h	r = 0.004
	P = 0.935
Cp2h	r = 0.048
	P = 0.351
Cp3h	r = 0.045
	P = 0.389
GLu0h	r = − 0.185
	P < 0.001
GLu0.5 h	r = − 0.252
	P < 0.001
GLu1h	r = − 0.238
	P < 0.001
GLu2h	r = − 0.216
	P < 0.001
GLu3h	r = − 0.185
	P < 0.001
HOMA-β	r = − 0.013
	P = 0.801
HOMA-IS	r = 0.192
	P < 0.001
MBG	r = − 0.21
	P < 0.001
SD	r = − 0.157
	P = 0.002
CV	r = − 0.076
	P = 0.142
LBGI	r = 0.133
	P = 0.01
HBGI	r = − 0.21
	P < 0.001
MODD	r = − 0.162
	P = 0.002
MAGE	r = − 0.092
	P = 0.078
ADDR	r = − 0.215
	P < 0.001

HbA1c hemoglobin A1C, TIR time in range, TBR time below range, TAR time above range, INS insulin, Cp C-peptide, Glu postprandial glucose, CV coefficient variation, SD standard deviation, LBGI low blood glucose index, HBGI high blood glucose index, MAGE mean amplitude of glucose excursions, ADDR average daily danger range, MODD mean of daily differences

### Multiple stepwise regression analysis of the factors influencing TIR, TAR, HOMA-β, and HOMA-IS

Multiple stepwise regression analysis was used to study the influencing factors of TIR, TBR and TAR, and the results showed that age, disease duration, TG, ALT, eGFR, osteocalcin, GluOh, Cp3h, and HbA1C were independent influencing factors of TIR (Table 3). The influencing factors of TAR and TBR were shown in Tables 4 and 5, respectively. Osteocalcin was found to be one of the influencing factors for HOMA-IS (ß = 0.188, P < 0.001) (Table 6), but not in HOMA-β (Table 7).

**Table 3:** Multiple stepwise regression analysis of influencing factors of TIR

	β	P	95%CI
(Constant)	117.31	0	96.762 to 137.859
Glu0h	− 2.279	0	− 3.028 to − 1.53
Cp3h	1.425	0	0.744 to 2.105
duration	− 0.592	0	− 0.907 to − 0.277
eGFR	− 0.119	0.001	− 0.188 to − 0.051
osteocalcin	0.532	0.007	0.144 to 0.92
TG	− 1.869	0.003	− 3.109 to − 0.63
Age	− 0.324	0.004	− 0.542 to − 0.105
ALT	− 0.171	0.022	− 0.317 to − 0.025
HbA1C	− 0.824	0.035	− 1.588 to − 0.06

Dependent Variable: TIR

**Table 4:** Multiple stepwise regression analysis of influencing factors of TAR

	β	P	95%CI
(Constant)	− 18.383	0.082	− 39.11 to 2.345
Glu0h	2.317	0	1.562 to 3.073
Cp3h	− 1.381	0	− 2.068 to − 0.695
duration	0.57	0	0.252 to 0.887
eGFR	0.124	0.001	0.054 to 0.193
osteocalcin	− 0.556	0.006	− 0.947 to − 0.164
TG	1.915	0.003	0.664 to 3.165
Age	0.311	0.006	0.091 to 0.532
ALT	0.172	0.023	0.024 to 0.319
HbA1C	0.866	0.028	0.095 to 1.637

Dependent Variable: TAR

**Table 5:** Multiple stepwise regression analysis of influencing factors of TBR

	β	P	95%CI
(Constant)	− 1.836	0.018	− 3.361 to − 0.311
Ca	0.502	0.016	0.095 to 0.909
Age	0.026	0.024	0.003 to 0.048

Dependent Variable: TBR

**Table 6:** Multiple stepwise regression analysis of influencing factors of HOMA- IS

	β	P	95%CI
(Constant)	1.861	0	1.215 to 2.506
BMI	− 0.026	0.006	− 0.045 to − 0.008
Osteocalcin	0.018	0	0.008 to 0.028
TG	− 0.046	0.005	− 0.078 to − 0.014
ALT	− 0.006	0.006	− 0.01 to − 0.002
SBP	− 0.004	0.01	− 0.008 to − 0.001

Dependent Variable: HOMA-IS

**Table 7:** Multiple stepwise regression analysis of influencing factors of HOMA-β

	β	P	95%CI
(Constant)	367.381	0.001	158.662 to 576.101
TC	− 51.553	0.019	− 94.685 to − 8.421

Dependent Variable: HOMA-β

## Discussion

Osteocalcin, a secreted protein consisting of 49 amino acid residues, plays a crucial role in the regulation of bone metabolism. However, recent studies cast new light on osteocalcin and suggest that bone can act as an endocrine organ involved in energy metabolism by secreting osteocalcin [20, 21]. Studies found osteocalcin levels were significantly lower in diabetic patients than in non-diabetic patients [22]. Hu found serum osteocalcin was also negatively associated with fasting plasma glucose in 2032 healthy Chinese women [23]. Higher OC levels were linearly associated with a decreased risk of diabetes in the non-diabetes subcohort [24]. Further research found that osteocalcin was negatively correlated with glucose concentrations in patients with different levels of glycemic tolerance, including normal glucose tolerance, impaired glucose tolerance and T2DM [25]. In the Swedish MrOS study, serum osteocalcin was considered as an independent negative predictor of plasma glucose [26]. Similarly, we found serum osteocalcin level was negatively associated with fasting and postprandial glucose concentrations at all time points and MBG.

High blood glucose variability is detrimental to the control and treatment of diabetes, an increase in dawn glucose level and glycemic excursions were negatively correlated with bone turnover markers [27]. To assess the relationship between bone turnover markers such as osteocalcin and glucose variability, CGM was used in this study to monitor blood glucose fluctuations and provide detailed information of glycaemic status. As an emerging metric derived from CGM data, TIR is commonly used to analyze the quality of glycemic control [7, 28]. Studies have shown that TIR describes not only short-term but also long-term glucose variability [29, 30]. Since TIR is highly correlated with HbA1C, TIR may be used to determine the outcome of clinical studies, predict diabetic complications, and assess glycemic control in individual patients [31]. A study of 3,262 diabetic patients confirmed the association between TIR and the incidence of diabetic retinopathy at all stages [32]. Using the DCCT dataset, Beck proposed that the hazard rate of development of retinopathy progression was increased by 64%, and development of the micro- albuminuria outcome was increased by 40%, for each 10 percentage points lower TIR [33]. A growing number of international organizations and guidelines support the inclusion of TIR as one of the target indicators for glycemic control and recommended that most diabetic patients have a glycemic control targets of TIR > 70%, TBR < 4%, and TBR < 1% [28].

In the present study, we found a positive correlation between serum osteocalcin and LBGI, while the relationship between osteocalcin and TBR had no significant correlation. TIR was significantly higher in the group with higher osteocalcin, while TAR decreased with increasing osteocalcin concentrations. In addition, various indicators of glycemic stability, such as SD, MODD, and ADDR were inversely correlated with osteocalcin, suggesting that osteocalcin may be a protective factor for TIR. Previous studies have shown that osteocalcin is positively correlated with HOMA-β [34] and negatively correlated with HOMA -IR [35]. We found that higher osteocalcin was a protective factor for HOMA -IS, whereas the relationship between osteocalcin and HOMA -β was not significant. Increasing insulin sensitivity and improving insulin resistance are crucial to maintain glucose homeostasis in diabetic patients [36]. Osteocalcin is able to increase insulin secretion or insulin sensitivity by stimulating the release of adiponectin [37–39], which is helpful to reduce blood glucose fluctuations. Osteocalcin could increase β-cell proliferation, energy expenditure, and adiponectin expression [40]. In vivo, administration of recombinant osteocalcin using subcutaneous mini-pump improved glucose tolerance and insulin sensitivity in mice [41]. Oral administration of osteocalcin exhibited similar effects [42].

On the other hand, constant high glucose could result in intestinal epithelial cell inflammation and apoptosis, which may be worsened due to drastic glucose fluctuation [43]. Anti-inflammatory medications may improve diabetes patient outcomes by reducing inflammatory activities and enhancing anti-inflammatory responses [44]. Recombinant LZ-8 dose-dependently reduced blood glucose, and haemoglobin A1c (HbA1c) by inhibiting inflammation and enhancing Tregs generation in diabetic rat model [45]. Likewise, osteocalcin can significantly reduce the secretion of pro-inflammatory cytokines and relieve inflammatory effects induced by hyperglycemic, through the PI3K/Akt/NF -kB signaling pathway [46, 47]. Futhermore, osteocalcin can stabilize blood sugar by interacting with muscle, fat and other tissues, but the exact mechanism remains obscure [48]. Further studies are needed to validate these issues.

There are some limitations in our study. Firstly, the patients in this study underwent CGM for 3 days instead of the 10–14 days which was recommended internationally [7, 49]. Secondly, osteocalcin exists in two forms, carboxylated and undercarboxylated osteocalcin [50]. However, we only assessed total serum osteocalcin due to technical reasonsin. Thirdly, we did not perform cell- or animal- based experiments to explore the possible mechanisms. Last but not least, this was a retrospective observational study, with a relatively small sample size. A larger multicenter, prospective study is needed to validate our findings in the future.

In conclusion, circulating osteocalcin is positively correlated with TIR and negatively correlated with MODD, ADDR, and MAGE. Osteocalcin may have a beneficial impact on glucose homeostasis in T2DM patients.

## Data Availability

The datasets used and/or analyzed during the current study are available from the corresponding author on reasonable request.

## Abbreviations

T2D: Type 2 diabetes
CGM: Continuous glucose monitoring
BMI: Body mass index
SBP: Systolic blood pressure
DBP: Diastolic blood pressure
TC: Total cholesterol
TG: Triglyceride
HbA1c: Hemoglobin A1c
TIR: Time in range
TBR: Time below range
TAR: Time above range
CV: Coefficient variation
SD: Standard deviation
LBGI: Low blood glucose index
HBGI: High blood glucose index
MAGE: Mean amplitude of glucose excursions
ADDR: Average daily danger range
MODD: Mean of daily differences
